# Busting the Breast Cancer with AstraZeneca's Gefitinib

**DOI:** 10.1155/2023/8127695

**Published:** 2023-12-04

**Authors:** S. Chemmalar, A. R. Intan Shameha, Che Azurahanim Che Abdullah, Nor Asma Ab Razak, Loqman Mohamad Yusof, Mokrish Ajat, Kim Wei Chan, Md Zuki Abu Bakar Zakaria

**Affiliations:** ^1^Department of Veterinary Anatomy, Veterinary College and Research Institute, Tamil Nadu Veterinary and Animal Sciences University (TANUVAS), Theni 625534, Tamil Nadu, India; ^2^Natural Medicines and Products Research Laboratory, Institute of Bioscience, Universiti Putra Malaysia (UPM), Serdang 43400, Selangor, Malaysia; ^3^Department of Veterinary Preclinical Sciences, Faculty of Veterinary Medicine, UPM, Serdang 43400, Selangor, Malaysia; ^4^Biophysics Laboratory, Department of Physics, Faculty of Science, Laboratory of Cancer Research (MAKNA), Institute of Bioscience and Material Synthesis & Characterization Laboratory, Institute of Advanced Technology, UPM, Serdang 43400, Selangor, Malaysia; ^5^Department of Companion Animal Medicine and Surgery, Faculty of Veterinary Medicine, UPM, Serdang 43400, Selangor, Malaysia; ^6^Department of Veterinary Preclinical Sciences, Faculty of Veterinary Medicine and Natural Medicines and Product Research Laboratory, Institute of Bioscience, UPM, Serdang 43400, Selangor, Malaysia

## Abstract

Breast cancer is the most common cancer diagnosed in women, and in 2020, there were 684, 996 deaths due to this disease. Epidermal growth factor receptors (EGFRs) and their respective ligands have been blamed for the pathogenesis and resistance to treatment in specific breast cancer cases. With EGFR having four homologues: EGFR1, EGFR2, EGFR3, and EGFR4, in-depth understanding of EGFR biology led to the discovery of small-molecule inhibitors and antibodies against this receptor. Gefitinib (GEF), a tyrosine kinase inhibitor of EGFR1, possesses a vast potential for treatment against breast cancer and is supported by a multiplicity of experiments. Unfortunately, in clinical trials, GEF did not show the outcomes expected with complete response and disease progress. This is due to incomplete understanding of the molecular mechanisms involved in EGFR signaling and endocrine sensitivity. Hence, additional in-depth experiments are needed regarding various molecular pathways and crosstalk pathways to comprehend GEF's action mechanism thoroughly in breast cancer patients. In this review, the role of EGFR in the development and pathogenesis of breast cancer and the pharmacokinetics and pharmacotherapy of GEF for the treatment of breast cancer have been elaborated. Nanomedicines synthesized with GEF have shown positive experimental response, paving a promising path for GEF against breast cancer.

## 1. Introduction

Cancer is described as a non-communicable condition that occurs as the outcome of amassed genetic alterations that disturb the physiological cell signaling pathways and those responsible for monitoring the cell cycle, mending of deoxyribonucleic acid (DNA), and programmed cellular death [1]. Nearly one in four cases of cancer among women worldwide is breast cancer [2]. In the year 2020, the Global Cancer Observatory (GLOBOCAN) estimated that the incidence of breast cancer was 2,261,419 (11.7%) and the mortality was 684,996 (6.9%), and concluded that it is the most common type of malignancy and the second leading cause of death in women [3]. These breast cancer cells result in changes in the primary tissue as well as invade and colonise various organs of the body, resulting in metastasis, constituting an enormous challenge in cancer treatment.

Breast cancer research has made significant advancements in the understanding of the epidermal growth factor (EGF) receptor (EGFR) gene family and EGF. Human breast tissue development and progression are being governed by the transforming growth factor-*α* (TGF-*α*)-EGFR autocrine pathway. EGFR and its ligand TGF-*α* and EGFR2 [4] were found to be overexpressed in many breast cancer cases and are correlated with meagre prognosis, resistance, or dearth of response to treatments with hormones [5–7]. Unrestricted expression of tyrosine kinases due to amplification, deregulation, or mutation is a trademark of malignancy. The defects in the cell signaling events and the response underlying tumor proliferation and metastasis could be made targets and would result in the therapeutic killing of those tumor cells. Epidermal growth factor receptor-tyrosine kinase inhibitors, also called EGFR-TKI, embody the molecular-focused cure employed against the multiplicity of tumors, including tumors involving the breast tissue [8, 9].

One may wonder why EGFR should be considered as a choice of cancer therapy. The fundamental reasons are as follows. Firstly, co-expression of increased quantities of EGFR and their respective ligands results in a transformed cellular phenotype [10, 11], in this context, cancer. Secondly, although EGFR is observed in typical epithelial cells, it is excessively expressed in many epithelial tumors including breast cancer [11, 12], which has been related to worse clinical output in several patients [10, 11]. Finally, studies were carried out with the monoclonal antibodies produced against EGFR, and the inhibitors synthesized against the activity of the tyrosine kinase of the EGFR suppressed the malignant cells' growth. The abovementioned studies lead scientists to design treatments to inhibit the EGFR and result in tumor destruction. It could be argued that tyrosine kinases are imperative in various signaling pathways taking part in physiological cellular function, but EGFR-TKI shows higher discernment for the tyrosine kinase of EGFR, with slight or inactivity against the rest of the kinases like ERK-2 and MEK-1 [13]. When employing molecular targeting therapeutics for breast malignancy, various roadblocks need to be cleared to achieve the desired outcome: (a) How to determine the patient's suitability for employing Gefitinib? (b) Is there a need to test for EGFR overexpression to employ Gefitinib? (c) Could the biological profiling of tumors predict the therapeutic response? and (d) How to define the therapeutic regimen for the breast cancer patient?

Nevertheless, only two therapeutic approaches have been widely used in clinical studies. They are (a) monoclonal antibodies/therapeutic antibodies (MAbs)—etuximab (Erbitux®), trastuzumab (Herceptin®), Osimertinib [14], Nimotuzumab [15], panitumumab (Vectibix®) [16], and Necitumumab [17]; (b) small-molecule inhibitors of EGFR tyrosine kinase (EGFR-TKI)—first generation: ZD 1839 (Iressa®, Gefitinib), Erlotinib (Tarceva®), and Crizotinib (Xalkori®) [6, 10]; second generation: Lapatinib (Tyverb®), Afatinib (Gilotrif®), and Dacomitinib [18]. Monoclonal antibodies exert their action by blocking the ligand from binding to the extracellular area of the receptor. On the other hand, small-molecule inhibitors exercise their effects at the intracellular domain of the EGFR, and due to their small size, these molecules can enter the cells easily. As an alternate or in conjunction with conventional chemotherapy, these types of targeted medicines could be employed for supra-higher benefits to a restricted number of designated patients with improved survivability, lowered toxicity, and improved value of life [19].

Gefitinib (GEF) is recognized for its crucial role in the therapy of non-small-cell lung cancer (NSCLC), and in 2015, it was accepted by the Food and Drug Administration (FDA) as the first-line therapy against tumors bearing the EGFR mutation and NSCLC [7, 20]. Even though drugs like GEF represent the new frontier in chemotherapy, knowledge and progress have a meagre impact on the therapy outcome, and conventional chemotherapy remains the mainstream cure for most breast tumor cases. This review will provide (a) an intense synopsis of the EGFR and their vital role in breast cancer, (b) the status of GEF in breast cancer research, (c) usage of GEF alone or in adjunct with other medicines routinely used for the chemotherapy of breast tumors, and (d) various types of nanomedicines developed with GEF.

## 2. EGFR Protein Kinase Family

**Figure 1:**
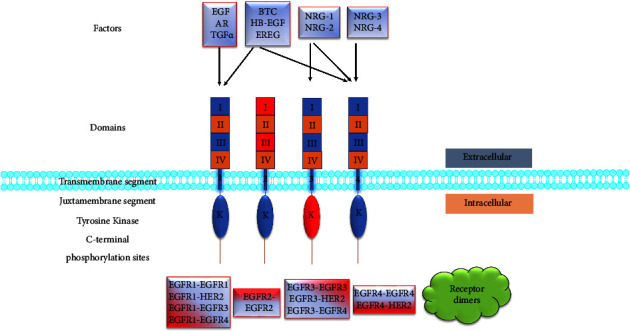
Binding specificities of EGFRs and preferred dimerization partners. EGFR 1, EGFR 2/HER2, EGFR 3, and EGFR 4 are represented by 1, 2, 3, and 4. Four categories of ligands bind to the EGFR family of receptors: epidermal growth factor (EGF), amphiregulin (AR), and transforming growth factor-*α* (TGF*α*) bind to EGFR-1; betacellulin (BTC), heparin binding epidermal growth factor (HB-EGF), and epiregulin (EREG) bind to EGFR1 and EGFR4; neuregulins, NRG-1 and NRG-2, bind to EGFR3 and EGFR4; and NRG-3 and NRG-4 bind to EGFR4. EGFR2 has no ligand binding [7, 25, 26], and EGFR 3 has a defective kinase (K) activity (red). After ligand binding, homo- or heterodimerization occurs. The most frequent heterodimers formed are EGFR2/EGFR4, EGFR1/EGFR4, and EGFR2/EGFR3 [27]. Adapted and modified from [22, 28].

One of the most intriguing and extensively researched growth factor receptors is the protein known as the epidermal growth factor receptor (EGFR/ErbB/HER) protein. The receptor family encompasses the four homologous receptors, namely, erbB-1 (EGFR1/HER1), erbB-2 (EGFR2/HER2/neu), erbB-3 (EGFR3/HER3), and erbB-4 (EGFR4/HER4) (Figure 1). EGFR is a transmembrane glycoprotein with cytoplasmic kinase activity that transduces growth signals from the extracellular site to the cell's intracellular environment [21]. These proteins belong to the type I subclass of the large family of receptor tyrosine kinases (RTKs), which share a greater percentile of homology in the sequence of the kinase domains. Tissues of mesenchymal, epithelial, and neuronal derivation and undifferentiated precursor cells express the EGFR [7, 22]. The gene coding for EGFR is situated on the short arm of chromosome 7 and it encodes a 170 kDa EGFR1 [19, 23]. The EGFRs in their extracellular region contain ligand-binding fields I and III except for HER2, which are leucine-rich, and domains II and IV contain multiple disulfide bonds. Domain II is involved in homo- and heterodimer formation. A single transmembrane hydrophobic (lipophilic) domain has about 25 amino acid residues and a cytoplasmic domain with about 550 amino acid residues that comprise a short juxtamembrane section, a protein kinase area, and a regulatory carboxyl-terminal segment [7]. The four EGFRs share a general structure of a pair of cysteine-rich sites in the extracellular area and a kinase site fringed by a carboxy-terminal end tail where tyrosine phosphorylation occurs [24]. Even though the EGFR family's affiliates share many functional and structural physiognomies, different phenotypes and variations in tissue expression in genetically modified mice indicate that each member of the family performs crucial functions, which remain unclear as to which member does what function [22]. EGFR signaling is crucial for cell multiplication and also contributes towards cancer progression, angiogenesis, metastasis [19], motility, adhesion, invasion, and inhibition of apoptosis [10].

### 2.1. Layered Signaling Pathways Triggered by EGFR Receptors

**Figure 2:**
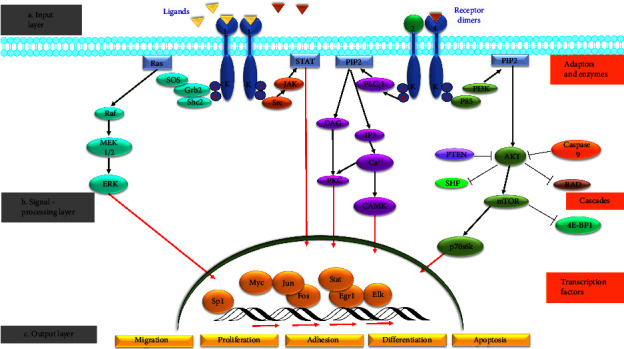
The layered EGFR signaling pathway. (a) Ligands and receptors comprise the input layer. The respective ligands and their receptors have already been shown in Figure 1. The numbers 1, 2, and 4 represent the EGFR1, EGFR2, and EGFR4, respectively. (b) Signal processing layer comprises the interactive adaptor/enzyme layer. Two receptor dimers: EGFR1-EGFR1 homodimer and HER2-EGFR4 heterodimer are shown. Shc2, SHC adaptor protein; Grb2, growth factor receptor-bound protein 2; SOS, son of sevenless; Ras, rat sarcoma protein; Raf, rapidly accelerated fibrosarcoma; MEK1/2, mitogen-activated protein kinase; ERK, extracellular signal-regulated kinase; Src, proto-oncogene c-sarcoma; JAK, Janus kinase/signal transducers; STAT, signal transducer and activator of transcription protein family; PLC*γ*, phospholipase C; PIP2, phosphatidylinositol 4,5-bisphosphate; DAG, diacylglycerol; PKC, protein kinase C; IP3, inositol 1,4,5-triphosphate; Ca^2+^, calcium cations; CAMK, calcium calmodulin-dependent protein kinase; p85, regulatory subunit of phosphoinositide 3-kinase; PI3K, phosphoinositide 3-kinases; PIP2, phosphatidylinositol 4,5-bisphosphate; Akt, serine/threonine kinases; PTEN, phosphatase and tensin homolog; SHP, src homology 2 domain-containing protein tyrosine phosphatase; Mtor, mammalian target of rapamycin; BAD, Bcl2 associated agonist of cell death; 4E-BP1, initiation factor 4E binding protein 1; p70S6K, p70 ribosomal S6 kinase. Only some of the pathways and transcription factors are represented in this layer. (c) Output layer is the end of the pathway which results in migration, growth, adhesion, or differentiation of the cell. Signaling pathways shown in the figure from left to right are Ras-Raf-MEK-ERK pathway, JAK-STAT pathway, PLC*γ*- PKC-CAMK cascade, and PIL3-AKT-mTOR cascade. Adapted and modified from [24, 31].

Knowledge regarding the EGFR signaling pathways is essential to understand their vital role in cell growth and tumorigenesis. The signaling pathway can be described in simple terms as follows: monomeric receptor tyrosine kinase units receive the ligand, activating the cytoplasmic catalytic action by inducing receptors to form dimers and subsequent auto-phosphorylation of tyrosine. These tyrosine residues act as docking sites for various enzymes, adaptors, or proteins, simultaneously recruiting various signaling cascades that result in the physiological response (Figure 2). All EGFR ligands are the targets of the Shc- and Ras-activated mitogen-activated protein kinase (MAPK) pathways . Majority of EGFR dimers have phosphatidylinositol-3-OH kinase [PI(3)K]-activated Akt pathway and p70S6K/p85S6K pathways [29], phospholipase C (PLCγ) [30] as the downstream pathways.

#### 2.1.1. Input Layer

The input layer consists of ligands and EGFRs. The epidermal growth factor- (EGF-) related peptide growth factors are the ligands that stimulate the EGFR receptors [32]. Ligands contain an epidermal growth factor-like site with three disulfide-bonded intramolecular coils [24], which confer binding specificity. According to their binding specificities, they are divided into four classes. The first class includes the EGF agonists; they bind to EGFR1 [6, 21], and this group comprises the EGF, transforming growth factor-*α* (TGF-*α*), and amphiregulin (AR). The second class contains Epiregulin (EPR), Heparin-binding EGF-like growth factor (HB-EGF), and Betacellulin (BTC) that exhibits dual specificity, hence binding to both EGFR1 and EGFR4. The third class comprises the neu differentiation factors (NDFs)/Heregulins/Neuregulins NRG-1 and NRG-2, which bind to EGFR3 and EGFR4 [33, 34]. The last class of ligands includes NRG-3 and NRG-4, which bind to EGFR4 (Figure 1) [35, 36]. With more than 25 ligands known [37], TGF-*α* is the prime modulator for cell multiplication in both healthy and malignant epithelial cells, and it is more potent than EGF [38]. EGF was demonstrated to be antiapoptotic in tumor cells excessively expressing EGFR, a condition observed in the vast majority of solid tumors [39]. According to research, TGF or EGF, which are produced by cancer cells themselves, can auto-stimulate EGFRs in cancer cells [19], which results in uncontrolled growth of cancer cells.

The biochemical features of the individual EGFR ligands control the signaling range. Firstly, the EGFR ligands are bivalent. Hence, they can form dimers, which in turn switch on various signaling pathways [32]. Secondly, biochemical property is their difference in binding affinities, which dictate the signal strength and interval. Thus, a low-affinity ligand could be a more effective signal inducer than their high-affinity ligands. Finally, the pH stability of ligand-receptor communication plays a dynamic role in receptor trafficking. EGF and its receptor interaction are fairly pH-resistant, whereas NRG-1 and TGF*α* quickly detach from their respective receptors at the endosomal pH, which results in the reprocessing of the receptor [40, 41].

Interesting information about the crucial horizontal network of interaction is that EGFR3 is lacking intrinsic kinase activity relative to EGFR1 as a result of replacements in important residues in its kinase region [42]. Due to this reason, despite having multiple ligands, it plays a role as a signaling unit when compared to other EGFRs. EGFR heterodimerization facilitates the incorporation of ligand-less EGFR2, which serves as a catalytic agent for a kinase-defective and preferred partner EGFR3, into signal transduction progression [43]. EGFR2-containing heterodimers are formed preferentially, which in turn potentiates EGFR signaling [43, 44]. A strict hierarchy is followed in heterodimerization, where EGFR2 represents the favored dimerization partner for all other EGFR receptors. EGFR2 is vital to laterally communicate initiating signals to EGFR3 [44] due to the specific binding choice for the bivalent ligands. The formed dimers are governed by the ligands and the cellular complement of EGFR receptors. EGFR2, when it forms heterodimers, subsequently demonstrates an increased ligand affinity as a result of “decelerated off-rate” [26] which is further linked to the extended stimulation of downstream signaling paths [45, 46]. On the contrary, the EGFR4 expression pattern is relatively restricted; various isomers of EGFR4 vary in their juxtamembrane and carboxyl-terminals; subsequently, there is a difference in the recruitment of PI(3)K [47], which is responsible for cell survival pathways.

#### 2.1.2. Signal-Processing Layer

The EGFRs exist as inactive monomers; when ligand-binding ensues, there is homo- or hetero-dimerization of receptors, followed by stimulation of intrinsic tyrosine kinase and auto-phosphorylation of specific C-terminal tyrosine residues [48] which provide docking sites for a protein containing Src homology 2 (SH2) or phosphotyrosine binding (PTB) domains [49, 50]. Tyrosine phosphatase 2 is a protein that is present in the Shp2 and Src homology domain-2. Through this recruitment followed by a series of intracellular events, this mechanism leads to the proliferation, sustenance, and even metastasis of cells.[5, 12, 51] (Figure 2). Numerous effector molecules bind to phosphotyrosine residues within the cytoplasmic site of EGFR kinases. These include Growth Factor Receptor Bound Protein 2 (Grb2), Grb7, Grb2-associatedbinding protein 1 (Gab1), and Shc; kinases like Src, phosphatidylinositol3-kinases (PI3K), and Chk through the p85 subunit. Protein tyrosine phosphatases like Shp1 and Shp2; Phospholipase C gamma (PLCγ); Cbl; and a few more selectively associate with specific EGFR kinases on particular phosphotyrosine residues [22] (for review, see [52]). As the adaptor proteins Grb2 and Shc [53] are bound to the receptors, they recruit SOS, which helps in the replacement of GDP with GTP in Ras, hence activating Ras. Activation of Ras leads to Raf's phosphorylation and, consequently, the activation of the MAPK pathway and the activation of numerous nuclear proteins, including cyclin D1, a molecule essential for advancement from the G1 to the S phase in the cell cycle [6].

Equally, interaction with Gab-1 and EGFR results in the enrollment of PI3K, which activates the AKT pathway by transmuting Phosphatidylinositol 4,5-bisphosphate (PIP2) to phosphatidylinositol (3,4,5)-triphosphate (PIP3). A negative feedback mechanism regulates these two processes. The activated ERK phosphorylates the SOS protein, resulting in the detachment of Grb2-SOS from the EGFR. Akt and PIP3 are further dephosphorylated by protein phosphatase 2 (PP2A) and phosphatase and tensin homolog (PTEN), respectively. Reactive oxygen species (ROS) is one of the vital stimuli for p38, which is a chief pro-apoptotic effector, and stimulation of neoplastic cells with EGF induces ROS production, hence activating p38. The dramatic decline of AKT and p-ERK with a delayed increase of p38 occurs due to the acute inactivation of EGFR. There is also evidence of favored variation in specific pathways since EGFR3 has several binding sites for p85 and it is the most effective activator of PI3K [54]. On the other hand, the epidermal growth factor receptor substrate (Eps15) gene and Cbl are specific for EGFR1 [55, 56]. Eps 15 and Cbl are responsible for the downregulation of the receptor EGFR1. Eps 15 binds to the clathrin adaptor protein AP-2 and also takes part in the coated-pit-mediated internalization [57].

EGFR1 molecules gather around clathrin-coated regions of the plasma membrane, which invaginate to produce endocytic vesicles, and these mature while gradually reducing the pH while accumulating hydrolytic enzymes, which results in receptor degradation. Hence, the rest of the EGFR receptors have weakened endocytosis and therefore more frequently recycled back to the cell surface [58]. The protein ligase essential for EGFR1 ubiquitylation and directing the receptor towards the lysosomal section is the Cbl, which is a RING finger area containing E3 ubiquitin [55]. Therefore, there is a drastic decrease in EGFR1 levels after ligand activation, and its activity is strictly ligand-dependent, a situation that is not observed in other EGFR3 and EGFR4, which have low kinase activity. In addition to receptor dimerization, the kinetics of specific pathways are also an essential factor. The cardinal process that turns off signaling by the EGFR system is the ligand-mediated endocytosis of receptors, and the kinetics of this process rest on the receptor's composition [24]. A highly regulated and dynamic equilibrium state exists between the activated, dimeric, monomeric, and inactive EGFR1 for the proper functioning of the cell, whereas on the other hand, when this mechanism is disturbed, then the equilibrium is disturbed, leading to destructive changes in the cell [59].

#### 2.1.3. Outcome Layer

The outcome of the signal ranges from cell proliferation and even migration (both related to tumor production) to cellular differentiation, adhesion, and programmed cellular death. The output layer is the end of the pathway that results in migration, growth, adhesion, or differentiation of the cell [24]. The cellular content, ligands, and the dimers formed are responsible for the variation in the output. While EGFR2 heterodimers were discovered to be the most potent complexes, homo-dimeric receptor combinations were found to be less mitogenic and transforming than the corresponding heterodimeric combinations [60].

### 2.2. EGFR-Driven Pathway and Cancer Development

A plethora of data exists that exposes the significance of EGFR receptors, especially EGFR1 and EGFR2, in human cancer progression. Enhanced transforming properties have been observed in cells expressing multiple EGFR receptors, the reason being the variety and signaling influence of the various EGFR receptor dimers [22]. Cancer cells have several mechanisms to trigger the network at various stages of the EGFR signaling pathway, that is, ligand overproduction, receptor overproduction, or constitutive stimulation of EGFR receptors [24]. Many cases of breast cancer were found to have overexpression of EGFR2 [61]. Consequently, it causes the kinase domain to become active, resulting in spontaneous dimerization. EGFR homodimers unaided may lead to cancer, but EGFR2 does form heterodimers, resulting in malignancy. The tumors that express EGFR2 also show autocrine stimulation of EGFR1 through its ligands [62]. The ability of the EGFR2 receptor to enhance EGFR1 signaling would increase growth stimuli and consequently stimulate additional intracellular pathways, maintaining the tumor's explosive rate of cell production. In the case of the human epithelium, the oncogenic capacity of EGFR2 may not depend solely on the presence of a specific ligand but mainly on its capacity to perform as a coreceptor for several stromal-derived growth factors [25]. Another EGFR that is co-expressed in overexpressing EGFR2 breast cancer is EGFR3 [63], and these cancers exhibit higher concentrations of phosphotyrosine on EGFR3 [64] due to the spontaneous dimerization of EGFR2 with EGFR3. These two receptors go hand in hand in stimulating the mitogenic signaling networks. Subsequently, cellular- Myc (c-Myc) and D cyclins become two important nuclear targets [65].

EGFR's role in the cell cycle and its role in transformed cells are different. EGFR does not typically retort to ligand stimuli in the M phase of the cell cycle; overexpression of the receptor detected in the tumor disturbs this cell cycle-dependent negative regulation [66]. Several mechanisms can be attributed to the instigation of the TGF*α*-EGFR autocrine growth pathway, like an accelerated expression of EGFR, an upsurge in the concentration of ligands, lessened receptor turnover, reduced phosphatase activity, and the incidence of aberrant receptors, which also includes EGFR gene alterations [6, 12].

### 2.3. Mammary Gland, Mutations, and Breast Cancer

The paired mammary gland in humans undergoes the utmost proliferation and differentiation postnatally. Under the stimulus of peptide and steroid hormones, the rudimentary system of ducts in the mammary gland undergoes tremendous development in adolescence. The breast tissue expresses all four EGFR receptors in developmental stage-specific patterns and cell types [67, 68]. Thus, in a nutshell, EGFR1 helps in the growth of the mammary gland, especially the growth of the ductal system, whereas EGFR4 and EGFR2 have a dynamic role in differentiation of the lobuloalveolar tissue and lactation [22]. The variety of signals from EGFR is due to the stock of ligands and the combinatorial properties of the dimers formed by the receptors. In various developmental phases of the mammary gland in mice, EGFR1 and Neuraminidase 1 (NEU-1) were found to be co-expressed. Luetteke and their team have generated mice with a targeted disruption of EGF, TGF*α*, and AR, in addition to triple null mice, and have found that there is functional redundancy in the role played by the EGFR ligands, and few ligands contribute specifically to the development of the mammary gland. Moreover, AR countenance was highest in the growing ducts, and specifically, deficiency of AR was related to the weakened growth of the ducts. The development of the lobuloalveolar region of the lactating mammary glands in AR null mice appeared typical, while in triple null mice, the process of lactogenesis was abolished [69]. Hence, it can be concluded that the mammary gland's functional differentiation involves the interaction of a multiplicity of EGFR ligands.

The protein tyrosine kinases have been found to have the largest portion of the dominant oncogenes, and in addition to this, EGFR and EGFR2, when activated by overexpression, mutation, or autocrine stimulation, are observed to have transforming effects in cell culture models and induce cancer in transgenic mice [70, 71]. The mutations in the EGFR have been spotted only in exons that encrypt the intracellular kinase domain [72]. Various studies to determine the role of EGFR in growth and development were carried out by analyzing the receptors of genetically modified mice, and it has been proven that null mutations observed in individual EGFR loci result in the lethality of the embryo or prenatal life, more specifically loss of EGFR leading to lethality with various abnormalities in organs including the skin, lungs, gastrointestinal tract, and brain [73, 74]. EGFR2 null mice were found to die during the mid-gestation period due to trabeculae malformation of the embryonic heart [75], which was similar to the observations in EGFR4 knockout mice [76]. All four types of EGFRs have been found to be expressed in the late pregnancy and early lactation glands [77]. EGFR is vital in the development of embryos, and it also plays an indispensable role in the growth and development of the mammary gland (Table 1). In the review by Samocha et al., the importance of the EGFR protein in the stroma and epithelial development of the mammary gland and its perturbations have been elaborated [78].

Excess sequence homologies and the existence of heterodimerization in the EGFR family members specify that the EGFR signaling pathway is non-linear, indicating the likelihood that other EGFR members besides EGFR could hold triggering mutations in human tumors [84]. Type III EGF receptor deletion-mutation (EGFRvIII) is the most common EGFR mutation in breast cancer [85, 86], and it plays a pivotal role in cancer progression [85]. The incidence of EGFRvIII expression in human invasive breast cancer samples was up to 67%, with normal breast samples showing negative expression, revealing the importance of the EGFRvIII mutation in breast carcinogenesis [87]. The gene rearrangement results in cancer cells overexpressing EGFRvIII. The salient feature is that the mutation results in abridged EGFR that do not contain domains I and II, so there is no ligand binding. On the contrary, this mutation has constitutively stimulated the tyrosine kinase domain, which accelerates cell multiplication independent of ligand interaction [6]. The deletion of exons 2–7 encoding the extracellular domains I and II is found in these mutated receptors [88]. In a study with EGFRvIII-transfected MCF-7 cells, expression of EGFRvIII increased EGFR2 phosphorylation through heterodimerization and crosstalk. In MCF-7/EGFRvIII cells, overexpression of the mutated receptor resulted in an increase in anchorage-dependent growth and accelerated colony formation in an anchorage-independent growth assay. The tumors formed from MCF-7/EGFRvIII cells were seven times larger than the control MCF-7 cells, proving the significant tumorigenicity of EGFRvIII [85]. In addition, co-expression of EGFRvIII with EGFR2 was found to amplify the downstream signaling events and enhance tumorigenesis *in vivo* [86]. It was also found that mutant EGFR, when activated, is more intense and prolonged in their activity compared to that of the activated wild-type receptor [72] and the mutant kinases are more sensitive to GEF, hence drug affinity.

*c-erbB* proto-oncogene encodes the EGFR, and the gene *neu* encodes for HER2 which has been found in various cancers. Amplification of HER2/*neu* gene has been associated with disease relapse and overall patient survival [4]. EGFR1 and EGFR2 receptors have been detected to play a pivotal role in the pathogenesis of human breast cancers [32]. Overexpression of EGFR1 has been observed in 14–91% (median value = 48%) of cases [62]. Overexpression of EGFR2 in the existence or absenteeism of gene amplification is observed in breast cancers [89], and higher levels of EGFR3 also have been demonstrated in primary breast cancer samples [90].

Breast cancer tissues were relatively autonomous of exogenous growth factors compared to the healthy breast tissue. This is due to the innate capacity of the tumor to synthesize increased quantities of growth factors and receptors for these growth factors [62]. Three main classes of breast cancer are human epidermal receptor-positive (HER2+), estrogen receptor-positive (ER+) or progesterone receptor positive (PR+), and triple-negative [91, 92]. Ductal carcinoma *in situ* (DCIS) is considered a phase 0 cancer, and it is a preinvasive breast lesion. If it remains untreated, it advances to invasive breast tumors in 25–30% of the cases. About 70% of the DCIS specimens were found to be the comedo subtype, which confers high proliferative, high mitotic index, ER, EGFR expression, and EGFR2 overexpression [23].

Breast cancers that do not express ER, PR, and HER2 are called triple-negative breast cancers (TNBCs). ER and PR-statuses can be defined as less than 1% of cancer cell nuclei testing positive by immunohistochemistry (IHC) [93]. TNBC expresses heterogeneity, and six types have been put forth: (1) basal-like 1 (BL 1), (2) basal-like 2 (BL 2), (3) immune-modulatory (IM), (4) mesenchymal (M), (5) mesenchymal stem-like (MSL), and (6) luminal androgen receptor (LAR). MSL subtypes showed enriched gene expression especially for epithelial-mesenchymal transition (EMT) and growth factor pathways including EGF, ERK½, PDGF, and G-protein coupled receptor (GPCR). BL 2 subtypes have gene ontologies involving growth factors (EGF, IGF1R, nerve growth factor (NGF), and Wnt/*β*-catenin) and growth factor receptors (EPAH2, EGFR, and MET). Many of these receptors and cognate downstream molecules are involved in the EGFR signaling pathway.

TNBC overexpresses EGFR and has activated downstream signaling pathways [94]. In the meta-analysis by Zhang et al., the expression level of EGFR was found to be statistically significantly higher in the TNBC than the non-TNBC [95]. In a study by Prat et al., where microarray was used to test breast cancer samples and cell lines, a significant increase in expression of EGFR1 was found in HER2-enriched TNBC as opposed to HER2-enriched/non-TNBC, which indicates that HER2-enriched tumors that are clinically not HER2-amplified could be governed by EGFR1. EGFR1 is involved in the pathobiology of non-basal HER2-enhanced TNBC, hence giving clinicians the choice of choosing a TKI for these types of tumors [96]. This can be understood by knowing that co-expression of EGFR and its respective ligands has been found in these breast cancers, which indicates that an autocrine loop mechanism functions in these cancers. Aberrant signaling by EGFR1 and/or HER2 has been spotted in breast cancer cases, and HER2 is also constitutively phosphorylated in these cancers since transmodulation occurred through the EGFR1 signaling [97].

In TNBC cases, higher EGFR copy number at the gene level has been associated with EGFR overexpression [98]. Gene amplification is responsible for this condition, and the EGFR copy number determined the prognostic value for poor disease-free survival in TNBC patients [99]. EGFR expression was higher in TNBC [100], EGFR overexpression leads to tumor cells receiving stimulus from cytokines, like heparin-binding-EGF, which are formed by the vascular endothelium, and the tumor cells are attached to the tissue with the help of selectins. Subsequently, there is augmented expression of cell adhesion molecules and intensification of tumor cell attachment to the walls of the blood vessel, hence assisting in the transmigration of these tumor cells to the extravascular areas [101]. This hematogenous spread of breast cancer cells, leading to metastasis in several steps, is facilitated by this process. Studies carried out in the MDA-MB-468 cell line indicated that EGFR controls cell-to-cell adhesion by modifying the interaction of actin in the cell's cytoskeleton and E-cadherin [102].

In a study by Kim et al., no activating EGFR mutations of the EGFR gene of clinical significance were detected in 148 TNBC cases, but five cases showed mutations in exon 19 or 21 [103]. Reis-Filho et al., in their study on metastatic breast cancer (*n* = 47), detected no activating EGFR mutations but found polymorphisms at codons 787 and 836 [104]. In European TNBC patients, there were no EGFR activating mutations detected in 229 patients [105]. On the contrary, mutations and single nucleotide polymorphisms have been detected in TNBC, where mutations were detected in exons 19 and 21 in 11.4% of the samples tested in Singaporean patients [106]. EGFR mutations at the gene level of 3.4% of TNBC cases have been detected [107]. EGFR gene amplification is correlated with EGFR overexpression in the study by Bhargava et al., but there were no gene mutations detected in the breast cancer samples. In the same samples, EGFR overexpression was well correlated with gene amplification [108]. In addition, positive EGFR expression did not correlate with the mutation in TNBC [106], stressing the importance of early diagnosis with molecular diagnostic methods to screen patients suitable for GEF.

EGFR2 deletions 755–759 were found to be homologous to EGFR exon 19 deletions that produced GEF-sensitive NSCLC, and consequently, this indicates that HER2 mutations would also influence cancer behaviour and outcome [109]. Furthermore, even at low concentrations, GEF was enough to inhibit these mutant receptors compared to the wild-type EGFR [53]. It is easier to select the NSCLC patients for treatment with tyrosine kinase inhibitors like GEF when compared with breast cancer patients. The mutations of EGFR observed in NSCLC were not consistently observed in TNBC cases, which directs us to follow protocols where the EGFR gene copy number [110], mutations in exon 19 and exon 21, and EGFR gene expression are needed to be assessed rather than only EGFR mutational analysis to define the patient's eligibility for GEF treatment. Moreover, the mechanism of EGFR overexpression in the absence of activating gene mutations is poorly understood [111]. As for using EGFR for the prognosis of cancer and/or for predictive aspects of treatment, there is a large amount of conflicting data, which is because there is variation in the laboratory techniques and non-uniform thresholds of positivity. In addition, there is heterogenous EGFR genomic instability in various breast cancer groups and ethnicity, and background also contributes to the disparity in the results [105, 106]. If there is a widely accepted criterion for EGFR status that is similar to HER2 status, there will be a large population of patients who will benefit from GEF as their first line of therapy for breast cancer [112].

### 2.4. Expression of EGFR1, EGFR2, EGFR3, and EGFR4 in Breast Cancer Models

The tumor microenvironment is different from the cell line cultures in the laboratory. The breast cancer cell line has similar genetic mutational alterations or DNA copy numbers, making them an apt tumor model. Cell lines are not true representative for cancer research since they do not have the heterogeneities of a true tumor. Moreover, cell lines expressing EGFR are more sensitive to GEF. Drug activity does not depend on the presence or absence of EGFR receptors, but several factors beyond target expression play a role in drug sensitivity [113]. The most crucial benefit of using a cell line is that it provides an unlimited number of relatively homogenous cell populations capable of self-replication in conventional cell culture media [114]. The breast cancer cell (BCC) lines are very vital *in vitro* models in the field of cancer research. SK-BR-3 and HCC1954 are breast cancer models for EGFR2 breast cancer. EGFR protein expression of commonly used breast cancer cell lines have been studied (Table 2). MCF-7 is one of the most commonly used BCC lines in research [115]. The SK-BR-3 cell line has amplified ERBb2 and high levels of EGFR, representing well-accepted model systems of ERBb2-positive breast cancer [116].

The development of new drugs and screening depend on the outcome of the cell culture studies, but the “one marker, one cell line [113]” has been questioned for a long time, taking into consideration the absence of heterogeneity, cell culture conditions, and culture microenvironment with alterations in the marker profile due to contamination or mutation of the concerned cell line used. However, the use of cancer cell lines expressing specific tumor markers is required for the initial research before validation using mouse models [115]. Genetically modified mice are those mice in which promotors could be employed to coax the expression of transgenes in the mammary epithelium, and many oncogenes have been expressed under the control of the promotors to initiate or moderate breast cancer development in mice. Mouse mammary tumor virus-polyoma middle tumor-antigen (MMTV-PyMT) and MMTV-Neu transgenic mice are two varieties of transgenic mice, where the expression of oncogene is through the mouse mammary tumor virus promoter, which brings about the development of malignant tumors [124]. MMTV-Neu mice have transgenic expression of the homolog of EGFR2. It is expressed at about 15 weeks postpregnancy, resulting in multifocal adenocarcinomas with metastasis to the lungs [125]. The MMTV-Neu mice have been a preferable model for EGFR-mediated mammary carcinogenesis. It was found that when rat mammary adenocarcinoma cells (MTLn3) were injected into the fat pad of mice, there was metastasis, which is indicative of the fact that EGFR acts by increasing the cellular motility and intravasation [126].

## 3. Gefitinib

**Figure 3:**
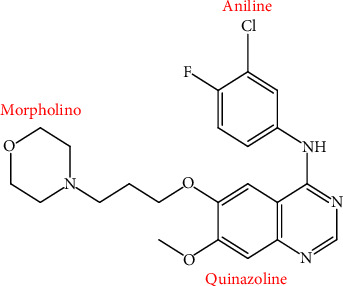
Chemical structure of Gefitinib. The anilinoquinazoline structure is responsible for tyrosine kinase enzyme inhibition.

AstraZeneca pharmaceuticals are famous in terms of their discovery and optimization of novel quinazoline-based compounds as anticancer drugs [127]. One of their innovations is ZD 1839, also called Gefitinib or Iressa [128]. It is one of the first-generation tyrosine kinase inhibitors. The timeline from discovery of GEF and milestones achieved reveal GEF to be a promising anti cancer drug (Table 3). GEF is a synthetic anilinoquinazoline [(4-3-chloro-4-fluoro-anilino)-7-methoxy-6-(3-morpholinopropoxy) quinazoline)] (Figure 3) with a molecular weight of 447 kDa [6, 21]. Iressa® has a structure similar to that of ATP, so it has more affinity for the ATP site as compared to ATP [127]; it demonstrates a triclinic crystalline structure [129]. The X-ray diffractogram and Fourier-transform infrared spectroscopy patterns of GEF have been studied sufficiently [130]. At pH 1, it is sparingly soluble, and with an increase in pH, its solubility decreases. Its solubility reduces in the upper gastric tract with its pH range of 4 and 6. This has an impact on the onset of action, bioavailability, and therapeutic capacity. The log *P* value of GEF is 4.15, which indicates that the drug is hydrophobic [131] and weakly basic [132].

### 3.1. Mechanism of Action of Gefitinib

**Figure 4:**
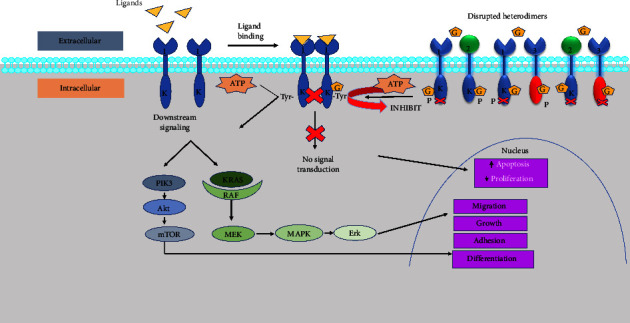
The mechanism of action and implications of GEF. GEF competes with ATP and binds to the adenine pocket of the active tyrosine kinase in the EGFR, hence preventing the downstream signaling and in turn the therapeutic effects of preventing the migration, growth, adhesion, and differentiation of cancer cells. The numbers 1, 2, and 3 represent the EGFR1, EGFR2, and EGFR3, respectively. PIK3, phosphatidylinositol-3-OH kinase; Akt, protein kinase B, serine/threonine kinase; mTOR, mammalian target of rapamycin; KRAS, Kristen rat sarcoma virus gene; RAF, rapidly accelerated fibrosarcoma kinases; MEK, MAPK, mitogen-activated protein kinase; Erk, extracellular signal-regulated kinase; ATP, adenosine triphosphate; G, gefitinib; K, kinase domain; Tyr, tyrosine. Adapted and modified from [30, 138].

The link between the altered EGFR gene and cancer has led to scientists developing targeted therapeutics against EGFR, which is easily accessible due to its vantage location on the cell surface. GEF is an effective type I reversible EGFR inhibitor that binds to the adenine-binding pocket of the active tyrosine kinase structure. The EGFR protein kinase domain comprises a small N-terminal lobe and a large C-terminal lobe, and these form a cleft, which aids as a site for attaching sites for ATP. *β*1 and *β*2 strands of the N-lobe dock with the adenine moiety of ATP and network with the GEF, thereby blocking the downstream signal transduction (Figure 4) [7]. The aforementioned EGFR mutations result in making the tumor cells more sensitive to GEF or any other EGFR-TKI, a phenomenon known as “oncogene addiction,” which justifies the rationale behind these molecular targeted therapies [27]. The inhibition has several effects, including cell cycle arrest, apoptosis induction, inhibition of invasion, metastasis, and finally, an increase in the antitumor effects of chemotherapy/radiation [143]. Figure 4 shows the mechanism of action and implications of GEF [138, 144]. GEF causes arrest at the G1 phase of the cell cycle, followed by apoptosis in the MDA-MB-231 cell line [94]. It targets the adenosine triphosphate (ATP) cleft inside the tyrosine kinase EGFR [13], induces cyclin dependent kinase (CDK) inhibitors p27 and p21, and decreases matrix metalloproteinases MMP2 and MMP9 enzyme activity. Most breast tumor cells show growth arrest by GEF, whereas only a subclass shows induction of programmed cell death [145], and higher quantities of the drug are required to induce apoptosis in healthy mammary epithelium and primary cultures of mammary carcinoma [146, 147]. The most important aspect of GEF is that it has no impact on the activity of serine-threonine kinases Raf, MEK-1, and ERK-2 (MAPK), with IC_50_ >10 *µ*M, >10 *µ*M, and >100 *µ*M, respectively, and these kinases also help in transmitting the EGF proliferative signals downstream of EGFR [13]. A similar study indicates that during a drug washout study with GEF, the inhibition of the EGFR was sustained for at least one day after a 2-hour drug exposure period [13], attributed to the drug sequestration in tumor cells whereby GEF sequestered EGFR into signaling inactive receptor-ligand complexes [148].

However, there is a wide range of levels of expression of EGFR in the various breast tumors, and no clear association has been detected between the level of expression of EGFR and the sensitivity to GEF [145, 147, 149]. This is because the extent of expression might not designate the extent to which cancer, or the cancer cell line, is reliant on EGFR signals for growth. Supplementary biomarkers that will precisely designate this necessity are still under research. The co-expression of other associates of the EGFR family which heterodimerizes with EGFR1 but whose signaling might be curtailed by GEF by its action on the EGFR element of such heterodimers might have a vital role in defining the sensitivity of GEF. GEF not only inhibits EGFR1 but also shows activity against EGFR2 at a 100-fold higher dose, as that is obligatory for EGFR1 inhibition [150]. When GEF is used for the treatment of EGFR2 overexpressing tumors, it results in dephosphorylation of EGFR2 and strong downregulation of PI3k/Akt signaling connected to dephosphorylation of EGFR3, which indicates the potential of GEF to be employed for EGFR2 overexpressing breast cancers in addition to EGFR overexpressing breast cancers. The inhibition of Akt activity is considered a marker for the sensitivity of breast cancer cell lines to GEF [37]. On the other hand, the Sh2 domain recognition sequence for the p85 regulatory subunit of PI3k has not been detected on EGFR or EGFR2, and p85 associates straight away with EGFR3, which comprises 7 repeats of the p85 recognition sequence [151]. EGFR3 was found to be expressed in most cell lines that were sensitive to GEF and could be pointed to as the reason for the PI3k activity in these cells. No correlation was found between sensitivity to GEF and EGFR3 and EGFR4 expressions. The studies using breast cancer cell lines overexpressing HER2 provide us with the result that GEF is selective for EGFR in vitro, but at the same time, in vivo, it reduces the basal phosphorylation of EGFR1, EGFR2, and EGFR3. But this activity occurs at a lower concentration of GEF and corresponds to antitumor activity. At IC_50_ of 1.2–3.7 *µ*M, GEF inhibits the EGFR2 kinase domain in vitro, in addition to EGFR1 [37]. This is because EGFR2 functions favorably as a heterodimer with other members, including EGFR1, resulting in receptor transphosphorylation and EGFR playing a vital role in HER2 expressing tumors. Moreover, the existence of somatic mutations in the EGFR1 gene in lung tumors correlates with the sensitivity of GEF [72]. GEF when taken orally, reversibly, and competitively prevents the binding of ATP in cancer cells. When inhibition of ATP binding to EGFR occurs, there is a block in the process of auto-phosphorylation and stimulation of downstream signaling pathways, resulting in reduced cell proliferation and apoptosis induction in tumor cells. The outcome is a quick decline of proliferative stimuli and a surge of pro-apoptotic signals in “oncogene addicted” tumor cells [27].

### 3.2. Pharmacokinetics

Deep insight regarding the pharmacokinetic profile of GEF in different organs is essential since the drug has an extensive tissue distribution (Table 4). The solubility of GEF is pH-dependent, and it has been found that food does not affect gastric absorption. Studies carried out on rats and dogs indicate that GEF had a bioavailability of only 60% [11], and it was also found that this drug was extensively distributed throughout the body. When GEF was administered orally as a single dose to healthy volunteers, it was determined that the peak plasma concentration (*C*_max_) was reached in 3 to 7 hours and *C*_max_ consequently declined biphasically [152], with a terminal half-life (*t*_1/2_) of 7.69 to 58.2 hours [153]. GEF followed linear pharmacokinetics. Extensive distribution in the tissues, except for skin and adipose tissue, and binding to alpha 1-acid glycoprotein (AAG) and human serum albumin (HAS) are the reasons for the high half-life. Metabolism is largely in the liver through cytochrome (CYP) 3A4 and to a lesser extent, the P-glycoprotein (P-gp) substrate [154]. The CYP3A4 inhibitors and inducers influence the pharmacokinetics of GEF (Table 5).

The majority of the whole drug (∼86%) and its metabolites (∼12.1%) were found to be eliminated in feces for over 10 days, whereas only less than 4% passed as such in the urine [11]. In a study where GEF was administered orally (250 mg/day for ≥14 days), the concentration of GEF was found to be 42 times higher than in plasma in nude mice bearing human tumor xenografts. GEF is metabolized by morpholine ring oxidation, and desmethyl-GEF (M523595) is produced by CYP2D6, which is the chief metabolite in plasma. It was found to be of similar concentration as that of GEF but not to have any therapeutic activity. In addition to this, M537194 was also found to be another metabolite of GEF [157].

### 3.3. Targeted Therapy with Gefitinib

Treatment of breast cancer is multifaceted, and it relies on the patient's health status, stage of cancer, and the availability and affordability of chemotherapeutic drugs. For advanced breast cancer cases, Pertuzumab, Trastuzumab, and taxanes like Docetaxel or Paclitaxel or Trastuzumab emtansine are used as the first- and second-line therapies, and then Trastuzumab or Lapatinib in combination with cytotoxic drugs can be employed as the third-line therapies. Not all the patients respond well to the current mode of treatment for breast cancer, especially when there is metastasis. Thus, there is a need of the hour to treat these patients with drugs that can be cytotoxic to hormone-dependent or independent tumor cells. GEF is one of the tyrosine kinase inhibitors used for the treatment of solid tumors [37]. It has proved to be an effective inhibitor of breast cancer proliferation, either unaided or in adjunct with other cytotoxic agents [9, 37, 97, 145]. Various studies have been carried out to determine the therapeutic outcomes of chemotherapy with GEF. The result of Phase I trials using GEF in patients was that it was very well tolerated, with the adverse events (AEs) being grade 1 to 2 gastrointestinal or skin events [159, 160]. GEF is administered orally to patients, which makes its intake flexible and convenient. It is also taken daily, which typically extends the exposure period of the tumor to GEF [161]. Studies indicate that GEF offers decent efficacy in Asian patients with EGFR mutation-positive cancer; hence, the mutation status of EGFR must be predetermined before treatment with GEF is commenced, particularly in a first-line clinical setting [162].

#### 3.3.1. Monotherapy with Gefitinib

Studies carried out on ZR-75-1 and MCF-10A ras breast cancer cell lines indicated that GEF (IC_50_ 0.2 to 0.4 *µ*mol/L) could impede colony formation in soft agar in a dose-dependent way, and the antiproliferative effects were found to be cytostatic. On the other hand, when the dose was increased, there was a doubled or quadrupled increase in apoptosis [13, 147]. GEF was also able to demonstrate effective disruption of EGFR2/EGFR3 interactions and was able to completely prevent TGF-*α*-induced mitogenesis of tumor cells expressing higher values of EGFR2 receptors [97]. Another study has revealed that GEF also has a probable role in tumor prevention, as it repressed the growth of xenografts derived from surgically removed DCIS of breast tissue, which were then implanted into nude mice,reduced proliferation along with increased apoptosis resulted after treatment with GEF [163]. This highly calls for future directions where high-risk patients with breast cancer could be treated with drugs like GEF for tumor prevention [13], demonstrating that mice bearing MCF-7 cell line xenografts responded to GEF treatment. Circulating tumor cells (CTC) in patients with metastatic breast cancer after treatment with GEF showed a decrease in the CTC in more than 50% of the patients. In addition, there was a significant decrease in the absolute number of EGFR1+/Cytokeratin+ and EGFR1−/Cytokeratin+ after the first month of treatment in 44.4% of patients [164].

Phase II trials of GEF monotherapy were conducted in two different patient groups: Group I (40) consisted of hormone-resistant HR-positive (ER+ and/or PR+ tumors) that showed tumor progression after treatment with both tamoxifen and aromatase inhibitors. Women in Group II (26) had HR (ER- and PR-) tumors. To explore the relationship between EGFR expression and disease control with GEF, 47 of the 66 patients enrolled in the trial had tumor samples examined for EGFR expression. However, a useful correlation could not be found because of the small number of individuals who saw a therapeutic benefit. This trial was closed prematurely due to the absence of a complete or partial response and the low clinical benefit rate [16]. Another phase II multicenter trial in 31 patients with previously treated, advanced breast cancer with GEF resulted in complete inhibition of EGFR phosphorylation found in tumor biopsies. But this trial resulted in no complete or partial responses, and it was concluded that the lack of significant clinical activity of GEF in these patients was not due to the lack of inhibition of EGFR but rather to the lack of EGFR dependence in these patients [165]. The results of the clinical trials were not promising, and hence research for further ways to improve the use of GEF was warranted.

#### 3.3.2. Combination Therapy with Gefitinib

In recent years, the hypothesis that combinations of targeted agents could show greater antitumor actions than their single-drug actions or their action in combination with conventional chemotherapeutic agents has led to clinical testing of combinations of targeted agents [166]. In addition, combination therapy came into use in chemotherapy because, in some instances, it overcomes drug resistance and disturbs multiple nodes of pathways of interest for a better outcome [167]. This has led to clinical success with multicomponent therapies [168] and multitargeted agents [169]. The main advantage of using a combination of agents with limited targets is that it can be tailored for individual regimes and unnecessary toxicities can be avoided [166]. Non-clinical evaluation of drug combinations utilizing appropriate *in vitro* cell lines and *in vivo* tumor models is essential to provide valuable insights into the mechanism of action of the drugs and their interaction in a combination.

An ideal justification for using GEF in combination with conventional cytotoxic drugs to achieve additive or synergistic anticancer effects is its mode of action and toxicity profile when compared to radiation and traditional cytotoxic drugs [170]. Phase II trials with advanced breast cancer patients indicated that no more than 10% of the patients responded to the GEF treatment [165], and these outcomes motivated the introduction of alternate modalities like combination therapy with cytotoxic agents or other co-targeting drugs or radiotherapy. The disadvantage of oral GEF intake is drug interaction. Drug-drug interactions could lead to adverse effects or reduced beneficial effects of either drug. The interactions could be due to pharmacokinetic and pharmacodynamic processes. The former arises when the absorption, distribution, metabolism, and elimination (ADME) of the involved drugs are changed, resulting in variations in the concentration and duration of the presence of drugs at the receptor sites. The latter refers to the interaction in which one drug changes the other drug's pharmacological effect [161]. This can be further classified as synergistic, additive, or antagonistic. Synergistic combinations are those combinations of drugs that result in an effect greater than the additive effects. H_2_ antagonists and proton pump inhibitors are not recommended to be used concurrently with GEF; when antacids are obligatory, they must be taken 2 hours before or after the intake of GEF [131, 155]. The salient studies using GEF in combination with other drugs have shown promising results (Table 6).

The synergistic combinations are used as the basis for many of the chemotherapeutic regimens, even used alongside radiotherapy or surgery. Phase I trials suggest that GEF can be administered to patients in combination with carboplatin and paclitaxel or leucovorin and 5-fluorouracil as a part of chemotherapy [171]. GEF prevents EGFR2 signaling in human breast cancer cell line that overexpress EGFR2 and cell lines which express functional EGFRs, by preventing EGFR1/EGFR2 heterodimerization [97]. In addition, breast cancer cell lines with low levels of EGFR expression were sensitive to GEF when they co-expressed high levels of EGFR2, and this effect is due to the GEF-induced reduction of EGFR1/EGFR2 heterodimerization [37, 97, 145].

In a phase II clinical trial with GEF and Cyclophosphamide combination therapy in estrogen receptor-negative early breast cancer patients, it did not increase the pathologic complete response (pcR) rate [51]. A phase II double-blinded, placebo-controlled multicentric trial on hormone receptor-positive breast cancer with combination therapy of anastrozole and GEF also yielded a similar result. This outcome could be due to an incomplete understanding of the molecular mechanisms involved in endocrine sensitivity [142]. In addition, research conducted in our lab shows synergism of GEF with paclitaxel in MCF-7 and SK-BR-3 cell lines (Chemmalar et al., unpublished work).

The meta-analysis of randomized controlled trials to determine the efficacy of GEF supplementation for breast cancer by Ye et al. concluded with the non-promising statement that GEF supplementation may not provide any positive effect on complete response, progressive disease, stable disease, or partial response for breast cancer patients. This meta-analysis was carried out on seven randomized controlled trials involving 927 patients. Additionally, it revealed that there was increase in adverse effects [175]. Though many combinations have been tested preclinically, many have not been tested clinically; furthermore, till date, no appreciable combinations have been found in clinical trials [8]. All these inconsistencies and failures could be due to an incomplete understanding of the molecular mechanism, and patients who have undergone adjuvant endocrine therapy could reduce the efficacy of GEF. Well-designed and controlled studies with molecular characterization of individual tumor samples along with baseline samples are required to further evaluate the possibilities of the treatment efficacy of GEF in breast cancer patients.

### 3.4. Resistance and Adverse Events (AE) of Gefitinib Chemotherapy

Resistance to GEF could occur due to the tumor's ability to utilize alternative growth factor pathways like insulin-like growth factor-I receptor signaling [176]. Conditionally activated EGFR (V-Erb-B : ER) is one of the reasons for drug resistance in breast cancer cells [177]. In TNBC, despite having overexpression of EGFR, the studies show that GEF lacks clinical efficacy. Various mechanisms result in resistance to GEF, including EGFR independence, EGFR mutations, and alternative downstream pathways. Activation of a bypass or downstream signaling pathways seems to be responsible for the resistance rather than the EGFR mutations in TNBC [8]. Some of the GEF resistant cell lines are shown in Table 2. The TNBCs are found to be not inherently sensitive to EGFR inhibition [178]. In addition, localization of EGFR to the lipid rafts contributes to resistance to EGFR-TKI [118]. The common AEs with a 250 mg dose of GEF, with exception to mortality, were skin and subcutaneous disorders (grade ½ skin rash and itching with dry skin), hepatobiliary disorder (elevation in alanine aminotransferase), gastrointestinal disorders (nausea, stomatitis, emesis, and diarrhea), metabolism, and nutritional disorder (anorexia) [51]. Grade ¾ AEs were found to be rare and were usually correlated with the underlying malignancy [12]. Patients experienced modest and temporary skin responses, such as rash or acne, since the basal layers of the epidermis express an elevated quantity of activated EGFR1 [19]. Another major AE observed in GEF treatment is QT_*c*_ interval prolongation. This has been associated with the interaction of TKI with hERG K^+^ channels, which results in an alteration in electrical flow and tardy conduction of the pulse [179]. This has been attributed to the chemical structure and plasma concentration of the drug. Even though this is a rare occurrence, there is a chance of prolongation of QT_*c*_ interval and subsequent development of Torsades de pointes which is a life-intimidating side effect [161]. The QT interval on an echocardiogram (ECG) represents the duration of the ventricular action potential, and QT_*c*_ is the corrected QT interval for the heart rate. According to the Bazett formula (QT_*c*_ = QT/√RR), RR is the time elapsed between two successive *R*-waves of the QRS complex [180]. Drugs that prolong the QT_*c*_ interval must be avoided, or if necessary, the ECG of the patient must be obtained 24–48 hours before and 1 week after the treatment [161].

### 3.5. Nanomedicines with Gefitinib

The biological and clinical trials with GEF showed inconsistency in its efficacy; hence, the usage of GEF in its “nano” form or loading it with a nanoparticle would reduce the overall systemic toxicity and help in delivering the medicine to the tumor site. The process of new drug development is not only time-consuming but also expensive. According to Walker and Newell, from 1995 to 2000, there were about 137 kinase inhibitors, and the overall phase I to registration attrition rates was 53% [181]. Nanoparticles are colloidal structures made up of organic or inorganic materials that are usually less than 200 nm in size [182]. Targeted drug delivery to a specific site is a sensible option to solve the issues faced in conventional chemotherapy. So, nanotechnology paves the way to solving all these critical issues by providing targeted therapy along with controlled drug release by delivering the payload to the targeted sites, hence reducing the toxicity [183, 184]. Nanotechnology can be used to improve the delivery of poorly water-soluble drugs, the co-delivery of two or more drugs for combination therapy, and the transcytosis of drugs across tight epithelial and endothelial barriers [185]. Moreover, nanoparticles have a higher surface area compared to their size which makes nanoparticles a perfect drug delivery system [186]. The major principle behind nanomedicine for the treatment of cancer is the enhanced permeability retention effect (EPR). Based on the observations that cancers have undeveloped lymphatic drainage and leaky vasculature due to continuing angiogenesis, it allows passive uptake of nanoparticles into the tumor [187].

The physicochemical characterizations: size, Polydispersive index (PDI), surface chemistry, drug loading and encapsulation, chemical composition, and impurities present, are required to be known to determine the stability and the biological effects of the synthesized nanoparticles [188]. The nanomedicines after they are formulated must be characterized stringently by following the guidelines of the FDA, and this is essential for research and development. The Investigational Drug Index (IND) application will give out the efficacy, toxicity, and physiochemical properties of the newly formulated nanomedicine. Only after approval from the FDA, human trials can be initiated so that the safety and efficacy of the new nanomedicine are determined. The FDA-approved categories of nanomedicines are micelle, metallic, protein, nanocrystal, liposome, and polymeric nanomedicines [189].

Among the various types of nanoparticles developed, many are very promising in being used for the treatment of cancer. There are a few nanodrugs formulated with GEF that have been synthesized in the laboratory (Table 7). *In vitro* and *in vivo* testing has been carried out only for a handful of the nanodrug delivery systems synthesized with GEF. All the abovementioned nanovectors pave way for a new era of treatment for breast cancer and other forms of cancer alike.

When compared to the conservative drug delivery systems, the drug-loaded nanoparticle/nanoparticulate system will more likely accumulate in the internal milieu of breast tumor because the size of these particles is in nanometers; this in turn helps in the enhanced concentration of drugs exposed to the tumor tissue, resulting in enhanced therapeutic efficacy. Another important fact is that the synthesized nanoparticles can encapsulate more drugs or a different class of drugs at once, without any interaction or decomposition. Compared to conventional therapies, this type of treatment has more advantages since the nanoparticles help improve the solubility of hydrophobic drugs by safely entrapping them and releasing them mainly at the tumor milieu in response to pH, as in Cockle shells derived calcium carbonate nanoparticles (CSCaCO_3_NP) [130]. By being loaded onto the nanoparticles, the drugs will be protected from degradation. Another point of consideration is that the drug release kinetics can also be adjusted depending on the different nanomaterials, size, and nanoparticles' structure [201]. Nanomedicine synthesized with TKIs like GEF has not yet reached the mainstream clinical trials, but a drug like GEF holds potential in combination treatment for certain TNBC and HER2 breast cancers.

## 4. Conclusions

Molecular targeted therapies provide us with a magic bullet type of drug that could be incorporated into treatment regimens for cancer patients, but still, it entails the assessment and monitoring of pathologic, genetic, and molecular markers that might or might not envisage a response to an explicit schedule. From the various research and trials carried out, it stands out that a positive association exists between EGFR and/or EGF family ligand status and various clinicopathological features of advanced tumor or grave prognosis. EGFR is an attractive and effective target on its own, provided it has a recognized role in cancer cell subsistence and spread. Although various trials have been underway, the clinical benefit of EGFR inhibitor therapy in breast cancer patients is still a mystery. In some cases, there is a prerequisite for the identification and clinical validation of useful norms for selecting patients who would potentially benefit from GEF. No doubt by far, GEF in combination with other drugs and nanomedicines will have an integral role in the battle against the dreadful breast cancer.

## Figures and Tables

**Table 1:** The various functions of the EGFR receptors during various stages of mammary gland growth and consequences of the mutated receptor.

EGFR receptors	Expression	Mice with mutated receptor
EGFR1	Expressed in entire phases of mammary gland development (mice with EGFR 1 mutation)	Scant development of mammary tissue along with defective ductal growth [79, 80]
EGFR2	Expressed in entire phases of mammary gland development (transgenic dominant-negative DN EGFR2 mice)	Ductal growth was normal, but lobuloalveoli were defective [81]
EGFR3	Essential during pubescent mammary gland development [77], enhanced lactogenic expansion and differentiation of mammary gland during pregnancy, by activation of Akt and STAT5A (transgenic mice expressing WAP-driven Cre recombinase) [82]	Ductal outgrowth penetration phenotype was defective, lobuloalveolar development was not affected (EGFR3-null glands in mice) [77]
EGFR4	Elevated levels especially in pregnancy and lactation (transgenic dominant-negative DN EGFR4 mice)	Impaired lactation [83]

**Table 2:** Salient characteristics of breast cancer cell lines in terms of EGFR expression.

Breast cancer cell lines	EGFR	EGFR2	EGFR3	EGFR4	Gefitinib sensitivity
MCF-7 [115]	Low levels [117, 118]	Low levels [117]	Low levels [119]	Naturally expressing [119]	Resistant
SK-BR-3	High abundance [120]	Overexpression [121], amplified [120]	Higher abundance [116]	Naturally expressing [44]	Sensitive [122]
HCC1954	High abundance [116]	Amplified [116]	NA	Not detected [116]	Sensitive [118]
T47D [32]	Low levels [117]	Low levels [117]	High expression	High expression	
MCF7/ADR [123]	Overexpression	Overexpression	NA	NA	Resistant
MDA-MB-231 [123]	Overexpression	Low levels	NA	NA	Resistant

EGFR: epidermal growth factor receptor 1; EGFR2: epidermal growth factor receptor 2; EGFR3: epidermal growth factor receptor 3; EGFR4: epidermal growth factor receptor 4; NA: not available.

**Table 3:** Milestones in the discovery and development of Gefitinib.

Year	Attributes
1960	Stanley Cohen isolated EGF from mouse submandibular gland [133]
1980s	Stanley Cohen discovered that EGFR has kinase activity [134], use of anti-EGFR antibodies [135], and discovery of EGFR-TKI having selective inhibition of tyrosine kinase activity [136]
1990s	Discovery of potent selective tyrosine kinase inhibitors of 4-anilinoquinazoline [127, 137]
2000s	Synthesis of Gefitinib [128], testing of efficacy of Gefitinib [13]
2002	Gefitinib approved for NSCLC [138], phase II clinical trials against breast cancer
2003	FDA approval of Gefitinib [139], clinical benefit in locally advanced/metastatic tumor with ER+ cancer
2010	Multicentric trial on postmenopausal women [140]
2013	Nanoparticles synthesized with Gefitinib [141]
2015	GEF accepted by the FDA as the first-line therapy against tumors bearing EGFR mutation and NSCLC [7, 20]
2016	Double-blinded, placebo-controlled multicentric trial on hormone receptor-positive breast cancer failed to show any clinical benefit [142]

**Table 4:** The pharmacological parameters of Gefitinib [154, 155, 156].

Gefitinib		
Target		HER1/EGFR/EGFR1

Chemical formula		C_22_H_24_ClFN_4_O_3_

Absolute bioavailability		60%

Protein binding		90%

Cytochrome P450 (CYP)	Major	Cytochrome P450 3A4 (CYP3A4), cytochrome P450 2D6 (CYP2D6)
	Minor	Cytochrome P450 1A1 (CYP1A1)
	Inhibits	CYP2D6, cytochrome P450 2C19 (CYP2C19)

**Table 5:** Effects of CYP3A4 inducers and inhibitor.

CYP3A4 inducer	CYP3A4 inhibitor	Changes in *C*_max_	Changes in AUC	Alternatives and recommendations
Rifampin		65% decrease	83% decrease	Increase the dosage to 500 mg per day [155, 158]
Phenytoin [154]		26% decrease	47% decrease	
	Itraconazole	51% increase	78% increase	Not recommended; if unavoidable, the adverse effects must be closely monitored [155, 158]

CYP3A4, cytochrome P450 3A4; *C*_max_, peak plasma concentration; AUC, area under the curve.

**Table 6:** List of salient combinations using GEF.

Drug combinations	Outcome
GEF + three different platinum-derived compounds (cisplatin, carboplatin, and oxaliplatin), two taxanes (docetaxel and paclitaxel), topoisomerase I inhibitor (topotecan), a thymidylate synthase inhibitor (raltitrexed), and two topoisomerase II inhibitors (etoposide and doxorubicin) [147]	Drug combinations against ZR-75-1 and MCF-10A ras show a dose-dependent supra-additive rise in inhibition of growth. GEF was cytostatic, but at higher doses and in combination with cytotoxic drugs, it augmented apoptosis.

GEF + (docetaxel, paclitaxel, or IDN 5109) [5]	GEF could restore sensitivity to taxanes (docetaxel, paclitaxel, or IDN 5109) in the MCF-7 ADR/bcl-2-overexpressing cell line and demonstrated a dose-dependent growth inhibition

GEF and herceptin, an anti-EGFR-2 humanized antibody	Combination therapy showed a frankly synergistic anticancer action *in vitro* and *in vivo* [9]
	Combination resulted in curtailing of the survival signal upregulated by EGFR signaling in EGFR-positive, HER2-overexpressing human breast tumor cells. GEF showed synergistic effects against SK-BR-3 and BT-474 cells [97].

GEF combined with either tamoxifen or Faslodex (ER downregulator) [172]	Synergistically enhance apoptosis and impede the cellular proliferation of the hormone-responsive MCF-7 cell line

GEF with tamoxifen [173]	MCF-7/HER2-18 cells showed elimination of crosstalk between the ER and HER2 signaling pathways

Phase II randomized, double-blinded clinical trials with 290 patients involved the combination of tamoxifen (20 mg/day per os) + GEF (250 mg/day per os) [174]	Resulted in adverse effects most common for the GEF drug, and the progression-free survival (PFS) median was 10.9 months in the group of women with newly diagnosed metastases or those who had recurred for a year or more after stopping adjuvant therapy with tamoxifen

A phase II clinical double-blinded multicentric trial on postmenopausal women with ER and/or PR+ with anastrozole (1 mg/day per os) + GEF (250 mg/day po) [140]	Resulted in some positive results with longer PFS for combination therapy compared to the patients receiving anastrozole or placebo

**Table 7:** Nanoformulations using GEF and its characteristics.

Nanoformulations	Characteristics
*Nanoformulations with GEF*:	
GEF-loaded PLGA nanoparticles [141]	Compared to GEF, exhibited higher anticancer activity on A549 lung carcinoma cells and A431 skin carcinoma cells
GEF-loaded poly (l-lactic acid) microspheres [190]	Nanoparticles were prepared by supercritical antisolvent (SAS) technology which were spherical, having a smaller and narrower particle size in comparison to GEF
GEF-loaded poly (lactide-co-glycolide) (PLGA) microspheres [191]	GEF-loaded polymeric microspheres were synthesized using an oil-in-water solvent evaporation method and wet-sieved to result in well-defined size fractions which corresponded to their characteristic drug release properties
GEF-loaded poly (ethylene glycol) 2000-distearoylphosphatidylethanolamine (DSPE-PEG2000) nano micelles conjugated with CD133 aptamers (M-Gef-CD133) [192]	First study to utilize nanoparticles to overcome the GEF resistance of lung cancer stem cells (CSCs) and could effectively deliver GEF to CD133^+^ lung CSCs
GEF-loaded blood cockle shells-derived calcium carbonate nanoparticles (GEF-CSCaCO_3_NP) [130]	GEF-CSCaCO_3_ NP synthesized using blood cockle shells (*Anadara granosa*) had a spherical shape with a diameter of 83.9 ± 28.2 nm and demonstrated zero-order kinetics with slow and sustained release
GEF on gold nanoparticles (AuNP) conjugated with EGFR antibody [193]	Synthesized nanoconjugates showed higher reduction in cellular viability in comparison with free GEF against A549, NCI-H460, and NCI-H1975 lung cancer cells after treatment for 48 hours
GEF-loaded poly(e-caprolactone)-poly(ethylene glycol)-poly(e-caprolactone) [194]	Compared to free GEF, the nanodrug has boosted antitumor effects and reduced toxic effects, and the survival time was prolonged in BALB-C athymic nude mice

*Nanoformulations of GEF with a combination drug*	
GEF-entrapped folic acid (FA) decorated bovine serum albumin (BSA) conjugated carboxymethyl-*β*-cyclodextrin (CM-*β*-CD) nanoparticles (FA-BSA-CM-*β*-CD NPs) [195]	FA-BSA-CM-*β*-CD nanoparticles were monodispersed and spherical and were able to induce apoptosis in Hela cells. These nanoparticles were internalised by clathrin-mediated endocytosis and macropinocytosis.
GEF and chloroquine-loaded chitosan NPs [196]	Co-delivery resulted in accelerated apoptosis and improved response in chemotherapy-resistant hepatocellular carcinoma (QGY) cell lines
GEF + paclitaxel- (PTXL-) loaded blood cockle shells-derived calcium carbonate nanoparticles (GEF-PTXL-CSCaCO_3_NP) [197]	GEF-PTXL-loaded blood cockle shell-derived calcium carbonate nanoparticles CSCaCO_3_NP synthesized without usage of toxic chemicals showed acceptable physicochemical characteristics being negative charged, spherical, mesoporous, and having zero-order kinetics of drug release
Folic acid- (FA-) conjugated GEF/capsaicin polymeric (PLGA-PEG) nanoparticles [198]	Synthesized for co-administration of GEF and capsaicin-loaded nanoparticles, and it resulted in a reduction of NSCLC tumor volume compared to treatment with individual drugs in albino mice. Significant downregulation of MMP9 and upregulation of caspase-3 and caspase-9 were observed in comparison to the individual therapy with GEF capsaicin.
Heavy chain Apoferritin/GEF (H-Aft/GEF) [199]	GEF in the H-Aft/GEF nanoparticles showed sustained release. Potent and improved antitumor activity was observed in EGFR2 expressing SK-BR-3 cell line when treated with H-Aft/GEF when compared with GEF alone (GI_50_ = 0.52 × 10^−6^ M versus GEF alone GI_50_ = 1.66 × 10^−6^ M at 120 hours incubation)
GEF/quantum dots-loaded peptide long-circulating liposomes [200]	Synthesized as a prognostic tool and a therapeutic agent for nasopharyngeal carcinoma which has resulted in increased drug uptake, with dose- and time-dependent cell growth inhibition

## Data Availability

No data were used to support this study.

## Abbreviations

AAG: Alpha 1-acid glycoprotein
ADME: Absorption, distribution, metabolism, and excretion
AEs: Adverse events
Akt: Protein kinase B serine/threonine kinase
AP-2: Activator protein 2
AR: Amphiregulin
ATP: Adenosine triphosphate
AUC: Area under the curve
BAD: Bcl2 associated agonist of cell death
BCC: Breast cancer cells
BL: Basal-like
BTC: Betacellulin
CaCO_3_: Calcium carbonate
Ca^2+^: Calcium cations
CAMK: Calcium calmodulin-dependent protein kinase
Cbl: Casitas B-lineage lymphoma gene
CDK: Cyclin dependent kinase
c-Myc: cellular-Myc
Chk: Checkpoint kinase
CYP: Cytochrome
CYP2D6: cytochrome P450 2D6
*C*_max_: Peak plasma concentration
CSCs: Cancer stem cells
CSCaCO_3_: Cockle shells derived calcium carbonate nanoparticles
CTC: Circulating tumor cells
DAG: Diacylglycerol
DCIS: Ductal carcinoma in situ
DN: Dominant negative
DNA: Deoxyribonucleic acid
ECG: Echocardiogram
EGF: Epidermal growth factor
EGFRvIII: Type III EGF receptor deletion-mutation
EGFR/ErbB/HER: Epidermal growth factor receptor
EGFR1/ErbB-1/HER1: Epidermal growth factor receptor 1
EGFR2/ErbB-2/HER2: Epidermal growth factor receptor 2
EGFR3/HER3: Epidermal growth factor receptor 3
EGFR4/HER4: Epidermal growth factor receptor 4
EGFR TKI: Epidermal growth factor receptor tyrosine kinase inhibitor
EMT: Epithelial-mesenchymal transition
EPAH2: Ephrin type-A receptor 2
EPR: Enhanced permeability and retention
Eps 15: Epidermal growth factor receptor pathway substrate 15
ER: Estrogen receptor
EREG: Epiregulin
ERK 1/2: Extracellular signal-regulated kinase
p-ERK: phosphorylated-Extracellular signal-regulated kinase
FDA: Food and Drug Administration
Gab1: Grb2-associated binding protein 1
GDP: Guanosine diphosphate
GEF: Gefitinib
GI_50_: 50% growth inhibition
GLOBOCAN: Global Cancer Observatory
GPCR: G-protein coupled receptor
Grb2: Growth factor receptor-bound protein 2
GTP: Guanosine triphosphate:
HAS: Human serum albumin
HB-EGF: Heparin-binding EGF-like growth factor
hERG: Human ether-a-go-go related gene
IGF1R: Insulin-like growth factor 1 receptor
IND: Investigational drug index
IM: Immune-modulatory
IC_50_: Inhibitory concentration for 50% of cells
IP3: Inositol 1,4,5-triphosphate
JAK: Janus kinase/signal transducers
KRAS: Kristen rat sarcoma virus gene
LAR: Luminal androgen receptor
*M*: Mesenchymal
MAbs: Monoclonal antibodies/therapeutic antibodies
MAPK: Mitogen activated protein kinase pathway
MEK1/2: Mitogen activated protein kinase
MET: Mesenchymal epithelial transition factor receptor
MMP: Matrix metalloproteinases
MMTV-neu: Mouse mammary tumor virus-neu with mutation of the transmembrane domain
MMTV-PyMT: Mouse mammary tumor virus-polyoma middle tumor-antigen
MSL: Mesenchymal stem-like
MTLn3: Rat mammary adenocarcinoma cells
Mtor: Mammalian target of rapamycin
NDF: neu differentiation factor
NGF: Nerve growth factor
NRG: Neuregulin
NSCLC: Non‐small‐cell lung cancer
NEU-1: Neuraminidase 1
PFS: Progression-free survival
P-gp: P-glycoprotein
pcR: pathologic complete response rate
PDI: Polydispersive index
PI(3)K: Phosphoinositide 3-kinases
PIP2: Phosphatidylinositol 4, 5-bisphosphate
PFS: Progression-free survival
PLC*γ*: Phospholipase C
PIP3: Phosphatidylinositol (3,4,5)-bisphosphate
PKC: Protein kinase C
PP2A: Protein phosphatase 2
p85: Regulatory subunit of phosphoinositide 3-kinase
PTB: Phosphotyrosine binding domain
PR: Progesterone receptor
PTEN: Phosphatase and tensin homolog
p70S6K: p70 ribosomal S6 kinase
p21: cyclin-dependent kinase inhibitor 1A
p27: cyclin-dependent kinases 1B
p38: mitogen-activated protein kinases
QT: Duration of the ventricular action potential
QT_*c*_: QT corrected for heart rate
Ras: Rat sarcoma protein
Raf: Rapidly accelerated fibrosarcoma
RING: Really interesting new gene
ROS: Reactive oxygen species
RTKs: Receptor tyrosine kinases
Shc2: SHC adaptor protein
SHP: Src homology 2 domain-containing protein tyrosine phosphatase
Src: Proto-oncogene c-Sarcoma
SOS: Son of sevenless
STAT: Signal transducer and activator of transcription protein family
STAT5A: Transgenic mice expressing WAP-driven Cre recombinase
TGF-*α*: Transforming growth factor-*α*
t1/2: Terminal half-life
TNBC: Triple negative breast cancer
ZD 1839: Gefitinib
4E-BP1: Initiation factor 4E binding protein 1.
